# First Report of *Klebsiella pneumoniae*-Carbapenemase-3-Producing *Escherichia coli* ST479 in Poland

**DOI:** 10.1155/2015/256028

**Published:** 2015-08-03

**Authors:** Dominika Ojdana, Paweł Sacha, Dorota Olszańska, Piotr Majewski, Piotr Wieczorek, Jadwiga Jaworowska, Anna Sieńko, Anna Jurczak, Elżbieta Tryniszewska

**Affiliations:** ^1^Department of Microbiological Diagnostics and Infectious Immunology, Medical University of Bialystok, 15a Waszyngtona Street, 15-269 Bialystok, Poland; ^2^Department of Microbiological Diagnostics and Infectious Immunology, University Hospital of Bialystok, 15a Waszyngtona Street, 15-269 Bialystok, Poland

## Abstract

An increase in the antibiotic resistance among members of the *Enterobacteriaceae* family has been observed worldwide. Multidrug-resistant Gram-negative rods are increasingly reported. The treatment of infections caused by *Escherichia coli* and other *Enterobacteriaceae* has become an important clinical problem associated with reduced therapeutic possibilities. Antimicrobial carbapenems are considered the last line of defense against multidrug-resistant Gram-negative bacteria. Unfortunately, an increase of carbapenem resistance due to the production of *Klebsiella pneumoniae* carbapenemase (KPC) enzymes has been observed. In this study we describe the ability of *E. coli* to produce carbapenemase enzymes based on the results of the combination disc assay with boronic acid performed according to guidelines established by the European Community on Antimicrobial Susceptibility Testing (EUCAST) and the biochemical Carba NP test. Moreover, we evaluated the presence of genes responsible for the production of carbapenemases (*bla*_KPC_, *bla*_VIM_, *bla*_IMP_, *bla*_OXA-48_) and genes encoding other *β*-lactamases (*bla*_SHV_, *bla*_TEM_, *bla*_CTX-M_) among *E. coli* isolate. The tested isolate of *E. coli* that possessed the *bla*_KPC-3_ and *bla*_TEM-34_ genes was identified. The tested strain exhibited susceptibility to colistin (0.38 *μ*g/mL) and tigecycline (1 *μ*g/mL). This is the first detection of *bla*_KPC-3_ in an *E. coli* ST479 in Poland.

## 1. Introduction

*E. coli* is a common etiological factor of urinary tract infection, gastroenteritis, neonatal meningitis, and many nosocomial infections such as pneumonia, bloodstream infections, and surgical site infections [1]. The treatment of infections caused by* E. coli* is challenging, because of the increasing resistance of bacteria to antibiotics. The phenomenon of multidrug resistance has been reported worldwide and results in reduction of therapeutic possibilities [2].

The aim of this study was to evaluate the presence of* bla* genes responsible for carbapenemases production (*bla*_KPC_, *bla*_VIM_, *bla*_IMP_, *bla*_OXA-48_) and genes encoding other *β*-lactamases (*bla*_SHV_, *bla*_TEM_, *bla*_CTX-M_). Additionally, we sought to determine the sequence type (ST) of a tested* E. coli* strain.

## 2. Materials and Methods

The tested* E. coli* strain was isolated in February 2014 from the swab of an intestinal fistula obtained from a patient hospitalized in the intensive care unit at the University Hospital of Bialystok (Poland).

Biochemical identification (GN cards) and the preliminary susceptibility test (AST-N259 cards) were performed using the VITEK 2 automated system (bioMérieux, France). Additionally, the susceptibility to antibiotics of the tested strain was performed using* E*-tests (bioMérieux, France). The results of the susceptibility tests were interpreted according to EUCAST recommendations [3]. The screening detection of carbapenemases was performed according to EUCAST. Moreover, the biochemical Carba NP test was performed according to the Nordmann and Poirel protocol [4]. Further, molecular analysis was performed with the use of polymerase chain reactions (PCRs). Plasmid DNA was extracted with the use of Plasmid Mini (A&A Biotechnology, Gdynia, Poland) according to the manufacturer's instructions. PCR amplifications for* bla* genes responsible for carbapenemases production (*bla*_KPC_, *bla*_VIM_, *bla*_IMP_, *bla*_OXA-48_) and genes encoding other *β*-lactamases (*bla*_SHV_, *bla*_TEM_, *bla*_CTX-M_) were performed using appropriate primers and conditions as described previously [5–8]. PCR amplicons were separated electrophoretically according to a previously described protocol [8]. Moreover, sequencing of* bla* amplicons was performed at Genomed (Warsaw, Poland). Multilocus sequence typing (MLST) was performed according to Institut Pasteur's MLST scheme (http://www.pasteur.fr/recherche/genopole/PF8/mlst/primers_Ecoli.html).

## 3. Results

The combination disc assay showed that the difference in the size of the inhibition zone between meropenem and meropenem with boronic acid was higher than 7 mm. The biochemical Carba NP test was positive after 1 minute. The obtained results indicated carbapenem resistance mediated by KPC among the tested strains of* E. coli*.

The tested strain was analyzed for the presence of resistance mechanisms against *β*-lactam antibiotics using PCR amplifications for* bla* genes responsible for carbapenemases production (*bla*_KPC_, *bla*_VIM_, *bla*_IMP_, *bla*_OXA-48_) and genes encoding other *β*-lactamases (*bla*_SHV_, *bla*_TEM_, *bla*_CTX-M_). The *bla*_KPC_ and *bla*_TEM_ genes were found in* E. coli*. The obtained sequence of the *bla*_KPC_ gene showed identity with the sequence of the *bla*_KPC-3_ gene (GeneBank accession no. AF395881.1). The obtained sequence of the *bla*_TEM_ gene showed identity with the sequence of the *bla*_TEM-34_ gene (GeneBank accession no. KC844056.1) responsible for production of broad-spectrum *β*-lactamase type TEM-34. Results of PCRs and minimum inhibitory concentration (MIC) values of tested antibiotics are presented in Table 1.

The analysis of allelic profile (*dinB-5*,* icdA-37*,* pabB-4*,* polB-10*,* putP-78*,* trpA-8*,* trpB-2*,* uidA-30*) with use of the* E. coli* MLST sequence type database (http://www.pasteur.fr/cgi-bin/genopole/PF8/mlstdbnet.pl?page=profile-query&file=Eco_profiles.xml) showed that the tested* E. coli* strain belonged to the ST479 type.

## 4. Discussion

A significant increase of* E. coli* isolates resistant to third-generation cephalosporins has been observed in Europe [9]. Studies have shown a high percentage (65%–100%) of extended-spectrum *β*-lactamase (ESBL) production among* E. coli* isolates resistant to third-generation cephalosporins [10]. One of the therapeutic options for treatment of infections due to ESBL-producing* E. coli* may be carbapenems. Resistance against carbapenems among* E. coli* rods is uncommon, which may be a result of AmpC *β*-lactamase production and loss of porins. Unfortunately, strains resistant to carbapenems due to the production of KPCs have recently been observed [11].

KPC producers have previously been reported in distinct geographic locations: European countries (Greece, Israel, Spain, Italy, Portugal, France, Poland, Germany, UK, and the Czech Republic), the United States, China, and South America [12]. KPC production is mainly prevalent among* Enterobacteriaceae* species. The significant majority of reports describe identification and the prevalence of *bla*_KPC_ genes among nosocomial* K. pneumoniae* strains. Moreover, the occurrence of *bla*_KPC_ genes among other* Enterobacteriaceae* species,* for example, E. coli*,* Enterobacter*, and* Citrobacter freundii* was observed [13]. The most commonly reported variant is KPC-2. Single reports describe the occurrence of KPC-3 among* E. coli* in Europe. In Spain, a multiresistant* E. coli* strain producing both KPC-3 and VIM-1 carbapenemases was described. In Italy, a KPC-3-producing* E. coli* isolate was found in abdominal drainage. Both cases were reported in 2014 [14]. Our study is first report of *bla*_KPC-3_ genes in* E. coli* ST479, in Poland.

## Figures and Tables

**Table 1:** MIC values of antimicrobial agents tested for *E. coli* 140 2594-2 and results of PCRs for *bla* genes.

Antimicrobial agents	MIC [*μ*g/mL]	
	Diffusion test with use of *E*-tests	VITEK 2 automated system and AST-N259 card
Amikacin	R 96	R ≥ 64
Amoxicillin/clavulanic acid	N	R ≥ 32
Cefepime	I 4	I 2
Piperacillin/tazobactam	R > 256	R ≥ 128
Cefuroxime	N	R ≥ 64
Cefotaxime	N	R 2
Ceftazidime	R > 256	R 32
Colistin	S 0.38	S ≤ 0.5
Ertapenem	R 8	R 4
Gentamicin	R 16	I 4
Tobramycin	N	R ≥ 16
Aztreonam	R 192	N
Imipenem	I 3	I 8
Meropenem	S 0.75	I 1
Doripenem	I 1.5	N
Tigecycline	S 1	S ≤ 0.5
Ciprofloxacin	N	R ≥ 4
Trimethoprim/sulfamethoxazole	N	R ≥ 320

Results of PCRs		
Genes encoding carbapenemases	Genes encoding other *β*-lactamases	

*bla*_KPC_-positive^*^	*bla*_TEM_-positive^**^	
*bla*_VIM_-negative	*bla*_SHV_-negative	
*bla*_OXA-48_-negative	*bla*_CTX-M_-negative	
*bla*_IMP_-negative		

R: resistant; S: susceptible; I: intermediate; ^*^genes encoding *β*-lactamase type KPC-3, ^**^genes encoding *β*-lactamase type TEM-34; N: not tested.
